# Understanding the Feasibility, Acceptability, and Efficacy of a Clinical Pharmacist-led Mobile Approach (BPTrack) to Hypertension Management: Mixed Methods Pilot Study

**DOI:** 10.2196/19882

**Published:** 2020-08-11

**Authors:** Lorraine R Buis, Dana N Roberson, Reema Kadri, Nicole G Rockey, Melissa A Plegue, Shivang U Danak, Timothy C Guetterman, Melanie G Johnson, Hae Mi Choe, Caroline R Richardson

**Affiliations:** 1 Department of Family Medicine University of Michigan Ann Arbor, MI United States; 2 Pharmacy Innovations and Partnerships University of Michigan Medical Group Ann Arbor, MI United States; 3 College of Pharmacy University of Michigan Ann Arbor, MI United States

**Keywords:** cell phone, mobile phone, hypertension, blood pressure, medication adherence, telemedicine, pharmacists

## Abstract

**Background:**

Hypertension is a prevalent and costly burden in the United States. Clinical pharmacists within care teams provide effective management of hypertension, as does home blood pressure monitoring; however, concerns about data quality and latency are widespread. One approach to close the gap between clinical pharmacist intervention and home blood pressure monitoring is the use of mobile health (mHealth) technology.

**Objective:**

We sought to investigate the feasibility, acceptability, and preliminary effectiveness of BPTrack, a clinical pharmacist-led intervention that incorporates patient- and clinician-facing apps to make electronically collected, patient-generated data available to providers in real time for hypertension management. The patient app also included customizable daily medication reminders and educational messages. Additionally, this study sought to understand barriers to adoption and areas for improvement identified by key stakeholders, so more widespread use of such interventions may be achieved.

**Methods:**

We conducted a mixed methods pilot study of BPTrack, to improve blood pressure control in patients with uncontrolled hypertension through a 12-week pre-post intervention. All patients were recruited from a primary care setting where they worked with a clinical pharmacist for hypertension management. Participants completed a baseline visit, then spent 12 weeks utilizing BPTrack before returning to the clinic for follow-up. Collected data from patient participants included surveys pre- and postintervention, clinical measures (for establishing effectiveness, with the primary outcome being a change in blood pressure and the secondary outcome being a change in medication adherence), utilization of the BPTrack app, interviews at follow-up, and chart review. We also conducted interviews with key stakeholders.

**Results:**

A total of 15 patient participants were included (13 remained through follow-up for an 86.7% retention rate) in a single group, pre-post assessment pilot study. Data supported the hypothesis that BPTrack was feasible and acceptable for use by patient and provider participants and was effective at reducing patient blood pressure. At the 12-week follow-up, patients exhibited significant reductions in both systolic blood pressure (baseline mean 137.3 mm Hg, SD 11.1 mm Hg; follow-up mean 131.0 mm Hg, SD 9.9 mm Hg; *P*=.02) and diastolic blood pressure (baseline mean 89.4 mm Hg, SD 7.7 mm Hg; follow-up mean 82.5 mm Hg, SD 8.2 mm Hg; *P*<.001). On average, patients uploaded at least one blood pressure measurement on 75% (SD 25%) of study days. No improvements in medication adherence were noted. Interview data revealed areas of improvement and refinement for the patient experience. Furthermore, stakeholders require integration into the electronic health record and a modified clinical workflow for BPTrack to be truly useful; however, both patients and stakeholders perceived benefits of BPTrack when used within the context of a clinical relationship.

**Conclusions:**

Results demonstrate that a pharmacist-led mHealth intervention promoting home blood pressure monitoring and clinical pharmacist management of hypertension can be effective at reducing blood pressure in primary care patients with uncontrolled hypertension. Our data also support the feasibility and acceptability of these types of interventions for patients and providers.

**Trial Registration:**

ClinicalTrials.gov NCT02898584; https://clinicaltrials.gov/ct2/show/NCT02898584

**International Registered Report Identifier (IRRID):**

RR2-10.2196/resprot.8059

## Introduction

Hypertension is a prevalent and costly burden in the United States, affecting about 116.4 million adults ≥20 years of age, and resulting in approximately $55.9 billion in estimated annual direct and indirect costs from 2014-2015. Poor rates of control exacerbate these concerns; of those affected, only about 50% of people achieve blood pressure control, and another 20% remain unaware of their condition [1]. Hypertension is a key risk factor for heart disease and stroke, which are the first and fifth leading causes of death in the US, respectively [2]. Thus, the identification of strategies to manage hypertension is vital to public health in the US. In 2017, the American College of Cardiology/American Heart Association implemented new hypertension guidelines defining hypertension as ≥130/80 mm Hg, lowering the threshold from the ≥140/90 mm Hg defined by the seventh report of the Joint National Committee on Prevention, Detection, Evaluation, and Treatment of High Blood Pressure (JNC7). As a result, the prevalence of hypertension among US adults increased from 31.9% to 45.6% [3], making this an even more salient health problem to address.

Clinical pharmacists, who assist patients in managing chronic conditions in primary care clinics [4,5], provide effective management of hypertension [6,7]. Another strategy for hypertension management is home blood pressure monitoring [8-12], although concerns about data quality and latency are widespread in instances where patients maintain paper-based logs for self-monitoring [13-15]. One approach to close the gap between clinical pharmacist intervention and home blood pressure monitoring is the use of mobile health (mHealth) technology. Since about 96% of American adults have a cell phone, 81% have a smartphone, and the rate of smartphone adoption is growing [16], mHealth interventions may be a viable technique to increase the efficacy of clinical pharmacist care and home blood pressure monitoring.

The current mHealth landscape is limited by applications that do not support bidirectional patient-provider communication or automatic transmission of electronic data from home blood pressure monitors in real-time. However, a bidirectional intervention that allows for the immediate upload of electronic data has the potential to increase the number of hypertensive patients a clinical pharmacist could assist, as well as improve the quality of blood pressure management for patients.

The goal of this study was to investigate the feasibility, acceptability, and preliminary effectiveness of BPTrack, a clinical pharmacist-led intervention that makes electronically collected data available to the pharmacist in real-time for hypertension management. The bidirectional intervention supports both home blood pressure monitoring and medication adherence for patients with uncontrolled hypertension. The study also aimed to understand barriers to adoption and areas for improvement identified by key stakeholders so that more widespread use of such interventions may be achieved, and further research can occur.

## Methods

The BPTrack study protocol has been described elsewhere [17]; however, key elements are summarized below. All methods used in this study were approved by the University of Michigan Institutional Review Board (HUM00105772).

### Study Design

We conducted a one-group design, pre-post pilot study of BPTrack, a clinical pharmacist-led mHealth intervention, intending to improve blood pressure control in patients with uncontrolled hypertension through a 12-week pre-post intervention. All patients were recruited from a primary care setting where they received treatment from a clinical pharmacist at the recruiting clinic site for hypertension management. Participants completed a baseline visit at the recruiting clinic, then spent 12 weeks utilizing the intervention at home before returning for a follow-up visit.

### Recruitment

Patient participants were recruited from a Family Medicine clinic associated with a large Midwestern academic medical center. The clinic site serves a majority blue-collar, underserved African American and Hispanic population, and hypertension is a commonly treated chronic disease at the site. Recruitment occurred through two primary procedures: recruitment flyers distributed by clinic staff to potential patient participants and targeted recruitment letters. Further details of these recruitment methods are described elsewhere [17]. All patient participants received $25 cash and were allowed to keep the Bluetooth-enabled blood pressure monitor (Welch Allyn Remote Monitoring Upper Arm Blood Pressure Device RPM-BP100), worth approximately $100, to incentivize study completion. Data from the trial portion of the study were collected between December 2016 and September 2017.

Stakeholder participants were recruited through the purposive sampling of individuals affiliated with the BPTrack program or health care providers for enrolled patients. Solicitation letters or emails were sent to medical assistants, physicians, a nurse, and the current and former director of the Family Medicine clinic site, as well as the study pharmacist who managed blood pressure care for patient-participants during the study.

### Eligibility Screening and Consent

All potential patient participants were screened for eligibility through a phone interview. Once deemed eligible, candidates were scheduled for the baseline visit, where written informed consent was obtained by research staff and baseline data collection commenced.

#### Inclusion Criteria

To be eligible for participation, patients had to be English speakers, ≥18 years of age, possess a smartphone compatible with the mobile intervention, have a diagnosis and history of uncontrolled hypertension (systolic blood pressure >140 mm Hg and/or diastolic blood pressure >90 mm Hg with repeated measurements), under the care of a primary care physician at the recruiting clinic, and taking at least one antihypertensive medication.

#### Exclusion Criteria

Exclusion criteria included age >65 years or previously established with a cardiologist or clinical pharmacist for hypertension management. Exclusions also applied to those who were pregnant, had existing medical conditions that would make blood pressure control difficult or required frequent hospitalization. Disqualifying conditions included resistant hypertension, steroid-dependent asthma or emphysema, cirrhosis or hepatic failure, stage C or D chronic heart failure, stage IV or V chronic kidney disease, and terminal cancer or ongoing chemotherapeutic or radiation therapy. Patients were also excluded if they had other serious medical conditions that would inhibit their ability to self-monitor their blood pressure, such as stroke or dementia.

### BPTrack Intervention

The BPTrack intervention consisted of two different mobile applications, one for the patient participant and one for the clinical pharmacist, developed by Tactio Health Group and customized from their TactioRPM Platform. BPTrack was the fully automated patient-facing smartphone app for iOS and Android, and BPTrack Pharm was the mobile application for iPad used by the clinical pharmacist. Together, these applications allowed real-time electronic home blood pressure monitoring and medication adherence tracking so that the clinical pharmacist had access to reliable and timely data. As the apps are not publicly available, participants were granted access by study staff after trial enrollment. The apps were provided free to users for use during the trial. Both applications have been described previously [17].

Patients were asked to measure their blood pressure using the provided blood pressure cuff and sync or manually enter the readings into the app. A written manual provided instructions on how to prepare for and properly obtain blood pressure measurements. Patients were encouraged in the written manual and as part of onboarding to reach out to study staff using the study hotline with any questions, comments, or to report any adverse events. The study manual also included safety instructions related to symptoms or repeat measurements indicating hypotensive or hypertensive emergencies.

The clinician-facing app provided the pharmacist with a dashboard view of all enrolled patients and a summary of their recent blood pressure readings, as well as individual page views for each patient, with full access to patient-generated blood pressure data. The clinical pharmacist was instructed to use their best clinical judgment in the interpretation of the BPTrack Pharm data and the appropriate clinical follow-up.

### Data Collection

Data were collected in a variety of ways to determine the feasibility and acceptability of this mHealth intervention. Data collection methods are described elsewhere [17] and summarized below.

#### Patient Surveys

Patients completed investigator-developed surveys at baseline and 12 weeks. The baseline survey collected demographics, health status, hypertension history, self-reported medication adherence and use, and other characteristics. The 12-week follow-up survey also collected self-reported medication adherence and use, as well as perceptions of feasibility, acceptability, and effectiveness of the BPTrack intervention. The baseline and 12-week follow-up survey both took approximately 10 to 15 minutes to complete.

#### Clinical Measures

Blood pressure and medication adherence, as measured by pill counts, were assessed by research staff in the clinic at baseline and 12 weeks. Blood pressure measurements were gathered using manual blood pressure cuffs by trained research staff. Staff also educated patients on best practices for measuring blood pressure, including sitting upright with feet flat on the ground, keeping the measuring arm at heart height, and not having moved for 5 minutes.

#### Patient Utilization of the BPTrack App

Patient utilization of the BPTrack app was documented in a variety of ways. Home blood pressure readings were extracted from the BPTrack Secured Cloud and analyzed for blood pressure trends, as well as compliance with self-monitoring protocols. As previously described, patients were asked to take their blood pressure three times per sitting, twice a day. Any text logs sent from within the app from the participants to the pharmacist were extracted as well.

#### Patient Interviews

To more fully understand patient participant perceptions of the BPTrack program, we conducted semistructured interviews at the 12-week follow-up. Semistructured interviews lasted 2-32 minutes.

#### Patient Participant Chart Review and Abstraction

We conducted a chart review to abstract data to document patients’ health care utilization during the study period. Data abstracted from patient charts included the visit date, diagnoses, type (phone, in-person, or secure messaging) and reason for the visit, location, whether the visit was with the pharmacist or another provider, medication changes, and blood pressure measurements taken during the encounter.

#### Stakeholder Interviews

Finally, we invited key stakeholders (physicians, pharmacist, clinic medical director, clinic nurses) to participate in semistructured interviews. These interviews focused on hypertension management, the use of mHealth for hypertension management, how BPTrack was (or could be) used within the clinic, perceived effects of BPTrack, barriers to BPTrack use and implementation, and suggestions for improvement of the program.

### Statistical Analysis

Descriptive statistics were compiled for patient characteristics, perceptions of BPTrack, self-reported medication adherence, blood pressure, pill counts, and app and health care utilization. Categorical data were displayed as frequencies and percentages, and chi-square tests were used for comparison. Systolic and diastolic blood pressures were expressed as mean (SD), and pre- and postintervention blood pressure means were compared using 2-tailed paired-samples *t* tests. The effect of clinical pharmacist contact, other health care utilization, and app utilization with changes in blood pressure levels were assessed using Pearson correlations. Available patient characteristics were assessed regardless of study completion; however, only participants who completed the study were included in the analysis of pre- and postmeasures.

Qualitative thematic analysis of patient and stakeholder interviews was performed [18]. We used the concepts of feasibility, acceptability, and effectiveness as a guiding framework for analysis. Two coders analyzed the patient interviews by coding all transcripts, reviewing line-by-line, and resolved initial disagreements to develop an initial codebook. We revised the codebook when clarifications were needed or new categories arose. Finally, we examined code patterns to identify themes related to feasibility, acceptability, and effectiveness. As a validity check, we searched for disconfirming evidence for each theme to challenge themes against the data.

## Results

We enrolled 16 patients in the BPTrack pilot study; however, one patient was immediately withdrawn after informed consent, but before collecting study measures, as the home blood pressure cuff did not fit the patient’s arm properly. Improper fit of the blood pressure cuff would have led to inaccurate home blood pressure readings. The remaining 15 patients had a mean age of 52.2 years (SD 6.0), and were predominantly male (53.3%; 8/15), married (66.7%; 10/15), employed (73.3%; 11/15), and had a high school or less education (46.7%; 7/15), an annual household income <$50,000 (46.7%; 7/14), and private insurance (60.0%; 9/15). On average, participants had been living with hypertension for 12.1 years (SD 11.2), and the majority were on two antihypertensive medications (53.3%; 8/15). See Table 1 for a complete list of participant demographics. On average, participants had a mean systolic blood pressure of 137.3 mm Hg (SD 11.1) and mean diastolic blood pressure of 89.4 mm Hg (SD 7.7) at baseline. Out of 15 enrolled participants, we lost 2 to follow-up, as we were unable to reach them to complete final data collection assessments (86.7% retention).

**Table 1:** Participant demographics (N=15).

Characteristic		Value	
Age, mean (SD)		52.2 (6.0)	
**Gender, n (%)**			
	Female	7 (46.7)	
	Male	8 (53.3)	
**Income, n (%)**		N=14	
	<50K	7 (50.0)	
	50K-100K	3 (21.4)	
	100K+	4 (28.6)	
**Race, n (%)**			
	White	10 (66.7)	
	Black	3 (20.0)	
	Other	2 (13.3)	
**Marital status, n (%)**			
	Married/living as married	10 (66.7)	
	Divorced/never married	5 (33.3)	
**Insurance, n (%)**			
	Private	9 (60.0)	
	Medicaid/Medicare	6 (40.0)	
**Education, n (%)**			
	High school or less	7 (46.7)	
	Some college/2-year degree	4 (26.7)	
	Bachelor’s degree +	4 (26.7)	
**Employment, n (%)**			
	Employed/self-employed	11 (73.3)	
	Retired/on disability	4 (26.7)	
**Baseline health** **, n (%)**			
	Very Good	2 (13.3)	
	Good	8 (53.3)	
	Fair	4 (26.7)	
	Poor	1 (6.7)	

### Feasibility

Across multiple sources of data, we found the use of BPTrack to be feasible within a primary care clinic.

#### Participant Utilization of BPTrack

**Figure 1:**
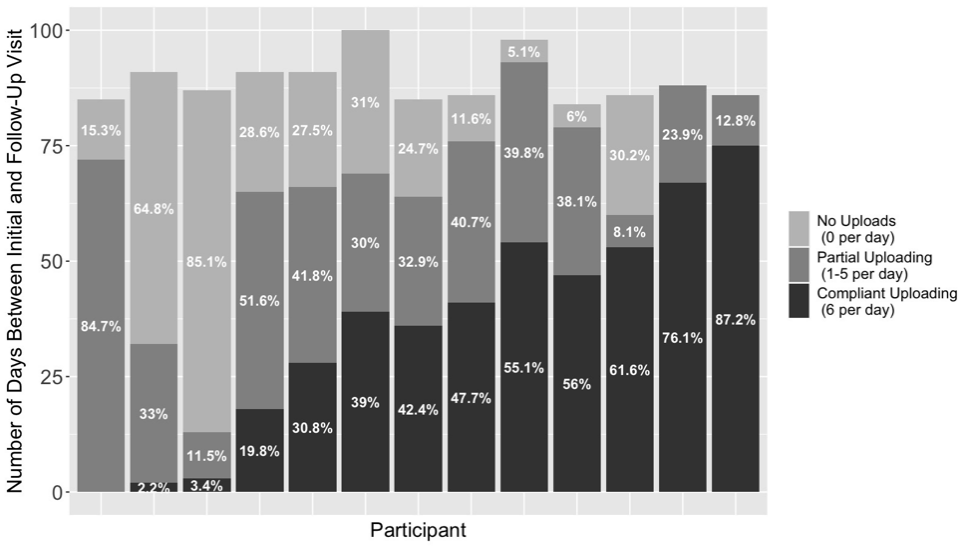
Patient adherence to blood pressure monitoring.

The BPTrack protocol asked participants to measure their blood pressure two times per day, with three measurements at each sitting, for a total of 6 daily measurements. All participants were given at least 12 weeks (84 days) between their initial and follow-up visits to upload data, and most uploaded well into the 12th week of the study period. Because not all follow-up visits occurred on day 84, participants had varying numbers of days in the study and were encouraged to continue to measure their blood pressure until the end-of-study visit. Use of BPTrack varied widely across the 13 participants who completed the study; the majority of people (n=11) uploaded at least one blood pressure measurement on 60 or more days, ranging between 13 and 93 days across participants (Figure 1). The average number of days uploading data was 66.4 (SD 22.2).

The number of measurements uploaded per day by any given person ranged between 1 and 12, with the average being 4.6 (SD 1.5). The most common number of measurements were 3 (27.7% of all days) and 6 (40% of all days). In terms of adherence to the monitoring protocol, we examined total compliance (6 measurements per day) as well as partial compliance (1-5 measurements per day) for all 13 completers. The number of days of total compliance for each participant ranged from 0 to 75, and the number of days where participants logged at least one reading ranged from 13 to 93 days. On average, participants uploaded at least one measurement on 75% (SD 25%) of study days. Also, on average, participants were fully compliant 40.1% (SD 28%) of the days between baseline and follow-up.

#### Participant Perceptions of Feasibility

Findings from patient surveys consistently showed that the use of BPTrack was feasible. The majority of participants agreed or strongly agreed that the program was easy to use (92.3%; 12/13), that learning to use BPTrack was easy (92.3%; 12/13), and that they tried to use BPTrack every day (92.3%; 12/13). Moreover, most patients did not find it challenging to incorporate the information from the application into their blood pressure management (92.3%; 12/13), and most patients did not find the integration of the blood pressure monitor and their cell phone to be confusing (84.6%; 11/13). Only 1 participant (7.7%; 1/13) indicated that using BPTrack took up too much of their time.

Findings from our semistructured patient interviews confirmed that patients viewed BPTrack overall as feasible for use within a clinical relationship. Generally, patients were able to utilize the BPTrack functions as designed, reporting, “I had no problems with the program” and “I just liked that it kept me informed. I could just open it up and look at it.”

Although patients saw BPTrack as a whole as feasible for use within primary care, two themes arose related to improving app function and adherence challenges. Patients recommended changes to the blood pressure cuffs, sync, and message functions to improve ease of use. While many patients reported that the blood pressure cuffs “worked well” and were “very sturdy,” one patient requested a larger cuff size and stated that the cuff tended to overinflate. Additional concerns expressed by multiple patients included trouble syncing or pairing their blood pressure cuff:

The… phone would unsync from the blood pressure. (Um-hm) And it would take several tries. Even though it said it paired, it did not pair. […] So I tried, you know, 5, 6, 7 times.

A few patients requested improvements to messaging. One suggestion was to allow custom messages:

Um, I had my own way of managing all that, you know. ‘Cause I have other things I like to remind myself of, and… to have yet another… set a notifications. And I turned it off. I, uh… I know you can’t do it in the app, but I can have, on Android I, you can, you know, force the notifications to stop. (Right) And I did. (Okay) So I, I turned ‘em all off.

Another patient pointed out that direct communication from the application to the medical record would be beneficial:

I’m not a reporting type person. So… it would be better for my health care, if…. It all just went straight to the medical record, and my doctor could just pull up my blood pressure reading.

Finally, several participants noted they had trouble adhering to the blood pressure monitoring protocol, which is not surprising given the BPTrack utilization statistics reported above. One concern was simply remembering to take blood pressure:

… sometimes it was… it was easy to, to forget to do it in the morning.

I did have to keep the monitor, like on a table, so that I would remember to do it, (Right) on a regular basis. I didn’t, if I didn’t see it, I wouldn’t think about it, so.

Patients also expressed concerns about keeping the routine for the long term:

I don’t know that I could take my blood pressure 3 times… every morning, and 3 times every night, for the rest of my life…”

It was kinda hard for me to sit down and take my blood pressure at 2 different times. But, so I did it all, l generally at the same time most days.

#### Stakeholder Perceptions of Feasibility

Despite our best efforts to recruit multiple stakeholders for semistructured interviews after the BPTrack pilot was complete, we only enrolled 1 pharmacist and 1 primary care physician. The stakeholders’ recognized the potential advantages of the BPTrack program. In particular, the physician indicated that “we have so many resources” and that BPTrack could “be part of the clinic workflow” with some minor adjustments. The physician even noted that something like BPTrack could “open up time for other stuff. I mean, we spend a lot of time… follow-ups, nurse visits for [blood pressure checks].” However, despite the feasibility and benefit of integrating BPTrack into routine practice, the stakeholders also indicated a few notable reservations, including the accuracy of readings, patient adherence, and a desire for data to transfer directly from BPTrack into the electronic health record (EHR).

In many ways, the physician’s and pharmacist’s perceptions of feasibility were similar. The physician explained, “I think it’s good to have, but I think we’re gonna see that it’s not… for everybody.” The physician indicated that better clinical decisions could be made when an accurate home blood pressure logs were available, either through a program such as BPTrack, or otherwise. Similarly, the pharmacist pointed out the utility of collecting the patient log in a mobile device: “It’s a very portable record because they almost always have their phone […] and they could share it with whoever they’re seeing.” However, they both raised concerns about accuracy. The physician pointed out the importance of using the cuff correctly: “Make sure the blood pressure cuff is… they’re doing it right at home.” Moreover, the pharmacist also noted concern with home blood pressure monitors in general: “I wish that they were more accurate; more consistent.”

Ensuring ease of use and minimal burden are critical to improving adherence. Regarding the application specifically, the physician explained:

It has to be very simplistic, especially for our older patients. So, I worry that if they have to type in their readings, that they’re gonna put… you know, wrong numbers.

The physician and pharmacist echoed patients’ concerns about the long-term sustainability of frequent monitoring. In terms of the desired frequency of home blood pressure testing, the physician and pharmacist suggested monitoring three times per week. The physician explained the potential burden of maintaining the twice-daily regimen long-term and suggested that “Ideally, 3 times a week… All at the same time [of day],” was more maintainable and useful.

“I think… like anything else it… when it starts, the patient might be very… motivated […] maybe at the beginning it’s really exciting, but after time people just… not really interested in using it.

Similarly, the pharmacist said: “I think once a day is awesome… if that’s asking too much, I think 3 days a week is usually sufficient.”

Finally, a concern was noted that the data from the application did not go directly into the EHR. The provider had to manually enter blood pressures and calculate averages, which was less than ideal. The pharmacist recommended:

I would love to be able to do that functionality, you know, just import that and have it… average them out for me.

The pharmacist went on to add:

The parts that we still need to work on are… getting that data into [the EHR]… Relying on a provider to log into a secondary system… long term is going to be very difficult.

Lowering the provider-side burden of managing the data will be necessary, mainly through integration with the EHR.

### Acceptability

#### Participant Perceptions of Acceptability

Patients self-reported a high degree of acceptability towards the BPTrack program with the majority indicating that they are satisfied with the program (92.3%; 12/13), the program was easy to learn (92.3%; 12/13) and use (92.3%; 12/13), and would like to continue using the program (84.6%; 11/13). Fully 100% (13/13) of participants who completed the follow-up assessment indicated that they liked being able to keep track of their blood pressures visually, they liked knowing someone was watching over their blood pressure in between clinic visits and would recommend BPTrack to others. Lending support for our pharmacist-led approach, only 23.1% (3/13) of participants agreed or strongly agreed that they would prefer that their doctor oversee their blood pressure. Only 7.7% (1/13) agreed that they had concerns about the privacy of their data.

Findings from our semistructured patient interviews confirmed that patients viewed BPTrack as acceptable for use within a clinical relationship. Thematic analysis revealed that participants were pleased with the program:

It was good. I enjoyed bein’ able to see… day to day what my blood pressure was and… you know, seein’ how I was improving, as I was takin’ the medication and stuff.

Another patient appreciated the convenience of checking blood pressure outside of a clinic:

Uh, the thing that I liked the most about the program was that it was in an environment that wasn’t at a doctor’s office… I could,… check my blood pressure in a usual state, either work, or home, or whatever. And I think that it gave a more accurate representation of what my blood pressure was like than to do it at a doctor’s office where I’m always… uh, stressed out.

Patients indicated they would continue to use the application if offered: “Uh, just that I, I would, if it was offered, you know, full time, I would, I would sign up for it.” Participants reported positive feedback regarding timeline and personalized blood pressure feedback:

It was… interesting. And… very informative, I thought, because I’m new to high blood pressure. And… um… it was neat to see the trends.

So without this app, I wouldn’t know… what my blood pressure was yesterday or the day before that…

A patient participant appreciated the timeliness of the information: “Before I… usually didn’t see my blood pressure till uh… the next, uh, visit, uh, to my doctor.” Participants also commonly reported the reassurance of having a health care professional monitor their incoming data:

And so I really… liked that part of it, where… it kept it recorded for me. And it was nice to know that it was… being read by a professional. You know, somebody that could help me if it did go out a whack.

Because of the increased monitoring by a health care professional, several participants reported that they had a more positive view of their medication regimen:

I just feel like I’m … uh, having, taking my medication based more on… on real blood pressure readings than a one or two-time visit to the doctor.

Despite positive views on the BPTrack program, participants noted a few areas for improvement. In particular, the daily medication reminders, as implemented, were not well-liked by all. Some complaints concerned the frequency of reminders:

The reminders were really nice at the beginning. But then… got annoying because, like I already know to take my blood pressure medicine at the same time every day. So the repetitiveness of it just got annoying.

Others suggested tailoring reminders and information beyond medication reminders:

I don’t know the messages were kinda you know, generic.

There’s a lot of little things that might get somebody to adjust their… their medication. But they only, um they only seem to address forgetting.

#### Stakeholder Perceptions of Acceptability

Both the physician and pharmacist indicated their acceptance of incorporating the BPTrack application into practice. The physician appreciated access to blood pressure readings:

(It) would be a benefit because… we could do a lot more pharmacotherapy management like that [and] if we can have that information available to us, then it should help us take better control of our patients.

In addition, the physician elaborated that extracting home blood pressure readings verbally from patients during office visits was time-consuming and often not representative of actual readings.

The pharmacist also appreciated access to readings, commenting that when logs are available, it was appealing to provide the patient with their data: “it’s a very cool, um… system to record blood pressures, so that patients have a record.” However, the pharmacist expressed reservations about timely reporting of elevated readings:

I wasn’t checking the app every single day […] I didn’t get enough, I guess, warning or that there was a problem.

### Preliminary Effectiveness

#### Effect of BPTrack on Blood Pressure and Medication Adherence

At 12 weeks follow-up, patients exhibited significant reductions in both systolic blood pressure (baseline mean 137.3 mm Hg, SD 11.1 mm Hg; follow-up mean 131.0 mm Hg, SD 9.9 mm Hg; *P*=.02) and diastolic blood pressure (baseline mean 89.4 mm Hg, SD 7.7 mm Hg; follow-up mean 82.5 mm Hg, SD 8.2 mm Hg; *P*<.001). Regarding medication adherence, at 12-week follow-up, the effect of BPTrack as measured by the Adherence to Refills and Medications scale was negligible and not significant (baseline 23.7 points, follow-up 23.1 points, *P*=.45). During this trial, 3 of our 13 participants had hypertension medications either removed from their treatment plan or had dosages lowered. Changes in systolic and diastolic blood pressure were not significantly associated with app utilization measures. There was a significant correlation between the number of hypertension-related encounters (*r*=0.77, *P*=.002) and the number of encounters with the clinical pharmacist (*r*=0.65, *P*=.02) and change in systolic blood pressure. In both instances, individuals with more encounters saw a greater reduction in their systolic blood pressure. There was no correlation with the number of encounters that were not hypertension-related (*r*=0.47, *P*=.11), nor was there an association with change in diastolic blood pressure and any of the health care utilization measures.

#### Participant Perceptions of Effectiveness

The majority of patients reported that using BPTrack was a benefit to their overall health (92.3%; 12/13), that BPTrack helped them to get their blood pressure under control (69.2%; 9/13), and helped them remember to take their medications (61.5%; 8/13).

Findings from our semistructured patient interviews confirmed that patients perceived BPTrack as useful in a variety of ways. Specifically, patients indicated that BPTrack helped raise their awareness of their hypertension and helped them to make behavior changes, such as eating healthfully, managing medications, and reducing stress.

Patients discussed how awareness helped them to consider what they were eating and make adjustments:

When I saw the different times of days and, and how that, how it varied across the day, um, you know, which got me thinkin' about what I, maybe what I was eating throughout the day and then the types of food.

A specific example was sodium intake:

It created an awareness within me to really pay attention to my diet, my salt intake, the types of food and, and become more involved with my overall health.

Despite some of the suggestions to go beyond medication reminders, some patients reported the program was helpful for medication management:

Helped me remember to take my medication for one thing.

Um, just heavy monitored. [The pharmacist] was able to make some adjustments, and we were able to delete some medication and increase some medication and get everything to a nice manageable level.

Other patients used the data to recognize the causes of stress by taking readings more frequently than office visits:

It’s very telling, I thought. I was able to… uh… distinguish a difference in my blood pressure, uh, based on my stress at work. (Um-hm) You know, when, when my work was more stressful, my blood pressure was definitely hanging out higher area, than it was, uh, when my work was less stressful… I never would have found out if I was taking my blood pressure manually or waited until I came to a doctor to have it taken.

Another patient explained being able to act more quickly to reduce stress:

It gave me the information [Systolic Blood Pressure] was 140, so I need to relax. It will be better after I get some rest. It kept me alerted as to what I should be doing. As opposed to not knowing at all what the blood pressure was. ‘Cause I felt the same the whole time through. I could never tell if my blood pressure was up or not.

#### Stakeholder Perceptions of Effectiveness

The physician and pharmacist both thought that BPTrack would have definite benefits when integrated into routine practice; however, both noted that solutions such as BPTrack cannot stand alone and need to be used in conjunction with standard care. While the physician felt it could be helpful, a concern was over-reliance on BPTrack to manage hypertensive patients:

Using this BP method, I worry that they won’t be seen for long periods of time. Or that we’ll… start throwing too many meds without having an adequate follow-up.

The physician also explained that regular visits are necessary and help the patient to feel more open to talking:

I still think patients should come in and be seen and, even if everything’s fine […] just a regular physical, making sure they’re taking their meds appropriately, that they’re not having any side effects, sometimes these things you won’t, they won’t tell you over the phone.” […] They’ll be more… likely to talk about their side effects or issues that they’re struggling with in person.

The pharmacist focused on how the application lent itself to improved patient education:

I liked that it graphs the numbers out for them, that it color-codes them. I think it helps patients learn, um, what’s good and what’s not good in terms of blood pressure control.

### Healthcare Utilization

Throughout the 12-week intervention, the 15 participants had 122 points of contact with their primary care clinic or the emergency department (mean 8 points of contact; range 4-20 points of contact). Of these points of contact, one involved a patient who presented to the emergency room experiencing symptoms associated with high blood pressure due to medication nonadherence and was treated accordingly. Most of the points of contact (78.7%; 96/122) included a hypertension-related focus (provider-initiated follow-ups to monitor BP, medications, symptoms, side effects, etc). These hypertension-related points of contact were conducted predominantly via phone (64.6%; 62/96), with in-person (33.3%; 32/96) and email (2.1%; 2/96) contacts occurring less frequently. Many of these points of contact are attributed to the increased blood pressure management oversight by our study pharmacist, as 76.0% (73/96) were contacts with our study pharmacist. Moreover, during the 12-week intervention, a total of 20 hypertension medication adjustments were made across our 15 participants (range 0-5 medication changes per participant), 70% (14/20) of which were made by the study pharmacist. Reasons for medication changes included adding or removing medications and dose adjustments.

The number of contacts with the study pharmacist (*r*=0.65, *P*=.02), number of hypertension-related encounters (*r*=0.77, *P*=.002), and number of encounters resulting in hypertension medication changes (*r*=0.68, *P*=.01) were all positively correlated with a change in systolic blood pressure. Given the high level of collinearity between clinical pharmacists and hypertension-related encounters and med changes as well as the small sample size adjusted analyses were not feasible.

## Discussion

Our pharmacist-led, mHealth supported approach to hypertension management shows great promise for helping to reduce blood pressures among uncontrolled hypertensive patients within primary care, as we have found this approach to be feasible, acceptable, and effective among patient and stakeholder participants.

### Feasibility

Like other recent digital health interventions for hypertension that use home blood pressure monitors, patients found BPTrack relatively easy to learn and use [19]. Complaints noted by patient participants largely related to issues that could be easily addressed through refining the app itself, as well as usage protocol. These issues include refinements to the BPTrack interface, documentation and protocols for the app and blood pressure cuff syncing, and the in-app messaging. Despite the burden placed on patient participants for self-monitoring, adherence to a minimum of daily self-monitoring was surprisingly high in this pilot, with participants self-monitoring their blood pressure at least once a day on 75% of the days in the study; however, criticism from patient participant concerning the self-monitoring protocol, which required three separate measurements twice daily, were common. Participants found the self-monitoring protocol to be laborious and time-consuming. Our decision to include such a rigid monitoring protocol was made because these were the monitoring guidelines in place at the University of Michigan at the time the study took place and followed guidance commonly recommended to patients by other institutions [20,21]. Interviews with health care providers revealed that despite those monitoring guidelines, they did not see the need to have that much data available, and the protocol would be likely laborious to patients. In reality, both providers agreed independently that self-monitoring blood pressure three times per week was sufficient. Furthermore, both providers noted concerns about the accuracy of home blood pressure cuffs, and regular calibration or comparison against clinic blood pressure cuffs was important to ensure the data was useful, with the pharmacist also noting that ensuring proper fit was essential. These are issues that have been established in the literature with clinicians expressing concern about home blood pressure monitoring and the use of inaccurate devices, adherence to protocols, or patient ability to interpret data [14]. As the use of remote blood pressure monitoring becomes more common within a clinical context, health systems should look to professional organizations such as the American Medical Association, for recommendations on how to engage patients in a way that maximizes data quality and patient care [22].

Providers were also united in their belief that tools such as BPTrack must integrate with other health information technology (IT) systems, namely the EHR, to be truly useful. As noted in several other studies, when systems contain patient data but do not integrate with the EHR, their usability is severely limited due to incompatibility with workflow [23-25]. In an ideal world, future research would seek to evaluate the efficacy of BPTrack or a BPTrack-like intervention, that is seamlessly integrated with the EHR to promote continuity of care and to leverage the collected data within the full care team.

As noted, the two participating providers suggested several concerns about the long-term feasibility of incorporating BPTrack or similar interventions into routine clinical practice. Our current clinical environment, and existing clinical workflows, are not designed to utilize digital interventions such as these. Most primary care practices in the United States do not have embedded clinical pharmacists, let alone the technological infrastructure needed to support this type of intervention.

In addition to reimagining workflow to support the expansion of digital health, well-designed protocols to address the inherent ethical concerns need to be established by health systems. For example, the pharmacist in the present pilot study expressed concerns about response times to concerning blood pressure values and a lack of a consistent alert system embedded in the EHR. Although the data was available to view, the pharmacist was unable to allocate time to review blood pressure values consistently. Integration into the workflow will become increasingly essential as mHealth interventions are scaled up. Also of concern is identifying the correct health care professional to review and respond to incoming patient-generated data. In the present study, we utilized a clinical pharmacist, but perhaps a medical assistant or personnel hired and trained to interact with this type of data specifically would be better suited [26].

### Acceptability

Qualitative and quantitative data reveal that patients found BPTrack to be acceptable for use. As noted, participants had high degrees of satisfaction with the program and would like to continue using the program. In particular, participants report that they found it valuable to see their blood pressure data, both daily as it was measured, and as a longitudinal trend. These observations are consistent with constructs such as self-regulation theory (on which this intervention is built) [17], which suggests that individuals engage in a dynamic feedback loop where they synthesize information about past behavior and integrate that information into goals and motivation to change future behaviors. Self-monitoring and self-reflection are key components of this dynamic feedback loop and are directly supported by the BPTrack intervention [27].

In addition to general thoughts on the acceptability of BPTrack, participants expressed their sense of satisfaction and safety in knowing their blood pressure measurements were being monitored by a health care professional who could make changes to their medication regimens. For some, this was noted as increasing satisfaction with the actual treatment plan, which may have downstream consequences for medication adherence [28]. For reasons of logistics, cost, reimbursement, and liability, many mHealth apps circumvent the health care system by focusing solely on consumer-facing apps; however, this pilot study demonstrates that individual patients have a real appetite to engage with their health care team through tools such as BPTrack rather than basic patient portals or EHRs. Likewise, on the whole, the two providers we spoke with agreed that there were benefits to engaging with patients through BPTrack for issues such as guiding treatment, although they were quick to point out concerns with an overreliance on the tool. Concerns were largely focused on implementation concerns (how to fit BPTrack into existing clinical workflow and health IT systems) as well as ethical considerations (issues related to liability and the responsibility to patients and their data). Regardless, expanded use of digital tools is likely to feature prominently in the future of health care, and implementation factors (including developing proper protocols to address ethical concerns) have been noted as the biggest hurdle facing expansion [26].

### Effectiveness

After 12 weeks, patients exhibited significant reductions in both systolic and diastolic blood pressure, both of which are considered clinically meaningful. Our finding of efficacy in this pilot study is supported by literature demonstrating the benefit of pharmacist-led [6,29-32] and self-monitoring interventions [32,33] for hypertension management. Not only were blood pressure outcomes improved at the end of 12 weeks, but patients’ perceptions of the effectiveness of BPTrack were noted in qualitative interviews.

Despite improvements in blood pressure and the presence of medication reminders within the BPTrack app, it is particularly interesting to note that our patients exhibited no significant improvements in medication adherence, which contributes to the already mixed literature on the effectiveness of mobile medication reminders [34-37]. Our adherence measures may not be sensitive enough to identify changes, which is a potential limitation of this study. It is also possible that medication adherence did not change, as we noted, but that participants engaged in other behavior changes that were not assessed as a result of their participation in BPTrack (increased physical activity or improvements in diet). Without knowing the mechanism through which participants managed to lower their blood pressure, it is difficult to identify how BPTrack led to improved blood pressure outcomes at 12 weeks, which is typical of black box interventions such as this.

### Limitations

This study was not without limitations. Given that the BPTrack intervention was available only to participants with smartphones, there are inherent digital-divide concerns. It should be noted though that smartphone adoption is exceedingly high (about 81% of American adults) and increasing; however, smartphone adoption is still lagging among individuals 65+ years of age (53%), with less than a high school education (66%), with annual household incomes <$30,000 (71%), and who live in rural areas (71%) [16]. Moreover, in the present study, our participants were quite heterogeneous in terms of socioeconomic status. Future work should seek to identify how this type of intervention fares with more disparate groups. Members of the study team are conducting a large randomized controlled trial of a home-based blood pressure self-monitoring intervention among uncontrolled hypertensive African Americans recruited from urban emergency departments and community settings (NCT02955537). Although this is not a pharmacist-led intervention, it does target a population that typically suffers from great health disparities, and who are more apt to be negatively impacted by the digital divide. This study was also limited by the research design, which was a simple one-group, pre-post design with no control group, as well as a short-term follow-up (12 weeks).

Moreover, the small sample size of both patients and stakeholders limits our findings. Future work should seek to look at longer-term implications, with larger sample sizes of patients and stakeholders, of a digital health intervention embedded within clinical care to determine whether the positive effects might be sustained over time. Finally, as with all packaged interventions, it is not clear which intervention components may have individually, or as a set, contributed to the positive reductions in patient participant blood pressure. Given that pharmacist-led interventions are efficacious [38-40], we do not know what the value add of the BPTrack app was. As we all desire to keep health care costs in check, future work should seek to understand whether the technology component added benefit above and beyond pharmacist-led interventions for managing hypertension that did not include technology, as well as to conduct cost-effectiveness studies of interventions like BPTrack, as well as pharmacist-led interventions without technology.

### Conclusions

Our results demonstrate that a pharmacist-led mHealth intervention that promotes home blood pressure monitoring and clinical pharmacist management of hypertension can be effective at reducing systolic and diastolic blood pressure in primary care patients with uncontrolled hypertension. Our data also support the idea that these types of interventions are feasible and acceptable to patients and providers and are effective at improving health outcomes. There is a need for more robust trials of digital health interventions integrated into routine clinical care to more robustly determine potential effectiveness, as well as guided investigations to more fully understand how to implement these types of interventions into clinical practice thoughtfully.

## Abbreviations

EHR: electronic health record
IT: information technology
mHealth: mobile health
